# Systemic inflammation and microglial activation: systematic review of animal experiments

**DOI:** 10.1186/s12974-015-0332-6

**Published:** 2015-06-06

**Authors:** Inge C.M. Hoogland, Carin Houbolt, David J. van Westerloo, Willem A. van Gool, Diederik van de Beek

**Affiliations:** Department of Neurology, Center of Infection and Immunity Amsterdam (CINIMA), Academic Medical Center, University of Amsterdam, Amsterdam, The Netherlands; Intensive Care Medicine, Leiden University Medical Center, Leiden, The Netherlands

**Keywords:** Microglia, Microglia activation, Systemic inflammation, Review, Animal experiments

## Abstract

**Background:**

Animal studies show that peripheral inflammatory stimuli may activate microglial cells in the brain implicating an important role for microglia in sepsis-associated delirium. We systematically reviewed animal experiments related to the effects of systemic inflammation on the microglial and inflammatory response in the brain.

**Methods:**

We searched PubMed between January 1, 1950 and December 1, 2013 and Embase between January 1, 1988 and December 1, 2013 for animal studies on the influence of peripheral inflammatory stimuli on microglia and the brain. Identified studies were systematically scored on methodological quality. Two investigators extracted independently data on animal species, gender, age, and genetic background; number of animals; infectious stimulus; microglial cells; and other inflammatory parameters in the brain, including methods, time points after inoculation, and brain regions.

**Results:**

Fifty-one studies were identified of which the majority was performed in mice (*n* = 30) or in rats (*n* = 19). Lipopolysaccharide (LPS) (dose ranging between 0.33 and 200 mg/kg) was used as a peripheral infectious stimulus in 39 studies (76 %), and live or heat-killed pathogens were used in 12 studies (24 %). Information about animal characteristics such as species, strain, sex, age, and weight were defined in 41 studies (80 %), and complete methods of the disease model were described in 35 studies (68 %). Studies were also heterogeneous with respect to methods used to assess microglial activation; markers used mostly were the ionized calcium binding adaptor molecule-1 (Iba-1), cluster of differentiation 68 (CD68), and CD11b. After LPS challenge microglial activation was seen 6 h after challenge and remained present for at least 3 days. Live *Escherichia coli* resulted in microglial activation after 2 days, and heat-killed bacteria after 2 weeks. Concomitant with microglial response, inflammatory parameters in the brain were reviewed in 23 of 51 studies (45 %). Microglial activation was associated with an increase in Toll-like receptor (TLR-2 and TLR-4), tumor necrosis factor alpha (TNF-α), and interleukin 1 beta (IL-1β) messenger ribonucleic acid (mRNA) expression or protein levels.

**Interpretation:**

Animal experiments robustly showed that peripheral inflammatory stimuli cause microglial activation. We observed distinct differences in microglial activation between systemic stimulation with (supranatural doses) LPS and live or heat-killed bacteria.

## Introduction

The peripheral immune system has a strong effect on the brain as exemplified by the high incidence of delirium and the strongly increased risk for the development of dementia after systemic infections [1, 2]. In rodent experiments, peripheral challenge with lipopolysaccharide (LPS) caused a steep increase of brain tumor necrosis factor alpha (TNF-α) that can persist for months [3–9]. Peripheral (systemic) LPS challenge activates microglia, the major active immune cells in the central nervous system. Microglia can be in a resting state (morphologically “ramified”) or an activated state (morphologically “amoeboid”) [10]. Resting microglia survey their environment for damage, ready to support endangered neurons or to interfere with a potential threat to the tissue integrity. Danger signals may trigger these surveying microglia and cause transformation to activated states, referred to as the M1 and M2 phenotypes [11]. M1 activated microglia produce pro-inflammatory mediators and are assumed to act as neurotoxic cells [11, 12], while M2 activation is induced by signals from apoptotic cells and have a role in remodeling and repair [11–13].

Sepsis in humans has also been associated with microglial activation [14]. Previously, we postulated that impaired cholinergic inhibitory control of microglia in elderly people, and to a greater extent in patients with (incipient) neurodegenerative disorders, contributes to uncontrolled neuro-inflammation [15]. High concentrations of pro-inflammatory mediators released by M1 activated microglia are potentially neurotoxic and might not only cause acute, reversible, behavioral effects, such as delirium, but also lead to persistent detrimental effects through bystander damage to neighboring neurons [16, 17]. The microglial response drifts out of control and ultimately causes neurodegeneration [1, 18]. This cycle might account for why neurobehavioral occurrences can persist in elderly patients after recovery from sepsis and after systemic cytokine production has fallen. This information inspired the formulation of a neuro-inflammatory hypothesis explaining the association of systemic infection, chronic central nervous system inflammation, and poor outcome, where microglial cells play a key role. Animal studies on systemic inflammation and microglial reaction support this hypothesis, but studies vary widely in setup and interpretation of results. In this review, we summarize available evidence on the effect of different systemic inflammatory stimuli on timing and intensity of the microglial reaction.

## Methods

### Search strategy

We searched PubMed between January 1, 1950 and December 1, 2013 and Embase between January 1, 1988 and December 1, 2013 for animal studies using peripheral inflammatory stimuli and evaluating the effect of these stimuli on microglia, using search terms “microglia” AND “animal model” NOT “review”. We also searched the reference lists of articles identified by this search strategy and selected those that we judged to be relevant. Two independent observers reviewed articles for inclusion and exclusion criteria, and differences were resolved by discussion.

### Selection of articles

Studies were included if they fulfilled the following criteria: (1) the study described an experiment where a peripheral infectious stimulus was administrated in animals, in vivo; (2) the study assessed the effects on microglia in the brain; (3) the effects on microglia were determined with specific microglial markers; (4) a group of control animals was described; (5) the study was an original full paper which presented unique data; and (6) the studies were published in English, French, or German. Reasons for exclusion of an article were as follows: (1) any manipulation in or around the brain before, during, or after the peripheral infectious stimulus; (2) the use of a neurotropic pathogen; (3) the use of a chemical synthetic infectious stimulus; (4) animal models in which the infectious stimulus reached the brain and caused secondary meningitis; (5) the use of transgenic animal models for a specific (brain) disease; and (6) animal models where the infectious stimulus was given intra-uterine. Full text articles of selected studies were obtained for further evaluation. Two independent observers extracted data and resolved differences by discussion.

### Data extraction

Each study was scored for key issues, such as animal species, gender, age, and genetic background; number of animals in treated and control groups; method of dosage, site of inoculation, and kind of infectious stimulus that was administrated; the effect of peripheral infectious stimulus on microglial cells in the brain; and methods of how this effect was determined on which time point after inoculation and in which region of the brain this effect was examined. The quality of studies was judged by a risk of bias assessment, scoring external and internal validity for each study [19].

### Definition of microglial activation

Microglial cells were defined as activated based on the following criteria: (1) microglia showed an activated morphology based on immunohistochemical staining; (2) there was a significant increase in number and/or size of microglia compared to the control group; and (3) there was a significant increase in expression of a microglial marker. When all three criteria were negative, microglia were inactive. If one or more criteria were positive, microglia were activated. If results were contradictory (e.g., increased expression of microglial marker but morphology was negative) microglia were judged as moderately activated.

## Results

### Description of studies

**Fig. 1:**
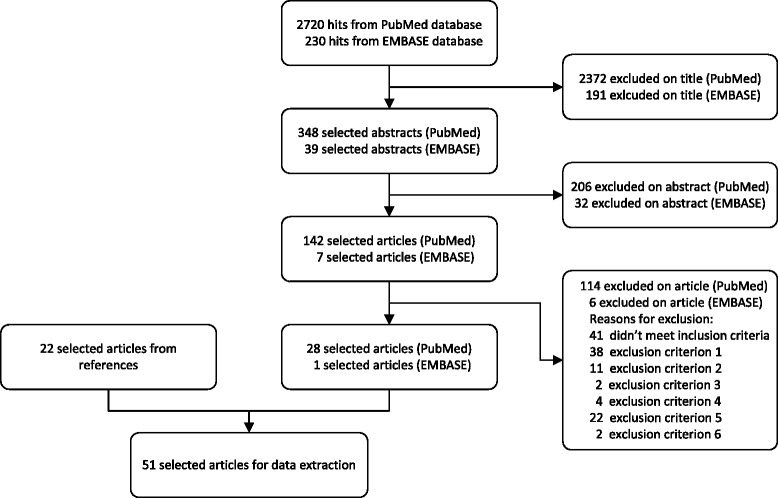
Study selection process

In total, 2950 publications were identified and 149 were selected for further review (Fig. 1); 29 publications met inclusion criteria and 22 additional articles were identified in a reference search, so 51 publications were included in this systemic review. There was considerable variation in animal species and stimuli. The majority of studies was performed in mice (*n* = 30) or in rats (*n* = 19). LPS was used as a peripheral infectious stimulus in 39 studies (76 %), and live or heat-killed pathogens were used in 12 studies (24 %).

Information about animal characteristics such as species, strain, sex, age, and weight was defined in 41 studies (80 %), and methods of the disease model were described in 35 studies (68 %). Four studies lacked description of animal characteristics and disease model. Just one study described treatment allocation as randomized and evaluation in a blinded fashion [20]. None of the studies provided power calculations for animal group sizes, reported baseline measurements of animals between groups, or handling of outlined or missing data.

### Outcome parameters

Microglial response was the main outcome parameter in all studies; the state of microglia was defined by immunohistochemistry in 36 studies (70 %), by the combination of immunohistochemistry, and respectively, quantitative polymerase chain reaction (qPCR) in two studies (4 %), Western blot in two studies (4 %), or in situ hybridization in one study (2 %); six studies used qPCR (12 %), three flow cytometry (6 %), and one study Western blot (2 %) to define microglial activation.

The most commonly used marker of microglial activation was the ionized calcium-binding adaptor molecule 1 (Iba-1), either as sole marker (*n* = 20), or in combination with cluster of differentiation 68 (CD68; *n* = 2), *Griffonia symplicifolia* isolectin B4 (IB4; *n* = 1), or macrophage receptor with collagenous structure (MARCO; *n* = 1). CD11b was used in 14 studies, and 5 of these studies combined CD11b with CD68, major histocompatibility complex II (MCHII), Toll-like receptors 2 and 4 (TLR-2, TLR-4), and F4/80. In three other studies, TLR-2 was stated as an activation marker of microglial cells after identifying the cells with CD45 and CD11b antibodies by flow cytometry or in combination with Iba-1. One study used TLR-2 with qPCR while MHCII expression between groups was similar [21]. The markers CD68 (*n* = 5), F4/80 (*n* = 2,) and IB4 (*n* = 2) were also used as sole markers.

The brain region of interest in the majority of studies was the hippocampus. Several studies restricted their interest to the hippocampal area (*n* = 17). Eight studies evaluated the hippocampal area in combination with other brain regions: cortex (*n* = 8), substantia nigra (*n* = 2), cerebellum (*n* = 2), thalamus (*n* = 1), striatum (*n* = 1), midbrain (*n* = 1). Five studies were limited to the cortical areas. Four studies homogenized the hemispheres for flow cytometry analysis or qPCR. The remainder 16 studies (25 %) evaluated other brain regions, and brain regions were not specified in one study.

Secondary outcomes were inflammatory mediators in the brain, for example cytokines, chemokines, Toll-like receptors (TLRs), or markers for damage or death, and were evaluated in 37 studies (73 %). Behavioral studies were performed in 14 studies (27 %).

### Infectious stimuli

#### Lipopolysaccharide

A single-dose of LPS was evaluated in wild-type mice in 20 studies (Table 1). LPS was administered intraperitoneally in 19 studies and subcutaneously in one study. Mice were male in 16 of 20 studies (80 %) and varied with respect to age and genetic background. The majority of studies used LPS derived from *Escherichia coli* (*E. coli*) (12 of 20 studies [60 %]), four studies used LPS from *Salmonella ssp*., and four studies did not report the LPS origin. The dose of LPS ranged between 0.33 and 200 mg/kg, with 1 and 5 mg/kg both used in seven studies. Microglial response was evaluated 1 h to 1 year after LPS injection.

**Table 1:** Single challenge with lipopolysaccharide (LPS) in mice and rats

Study	Genetic background	*N*	Age/weight	Sex	Type of LPS	Site of LPS	Dose (mg/kg)	Time of termination	Microglial activation
Mice									
Henry [33]	BALB/c	6	3 m	M	*E. coli* (O127:B8)	ip	0.33	1 day	Yes
Henry [21]	BALB/c	7	3–4 m	M	*E. coli* (O127:B8)	ip	0.33	4 h	Yes
			18–20 m					4 h	Yes
Carnavale [58]	C57BL/6	4	12–15 w	M	*S. equine abortus*	ip	0.5	1 day	Yes
								5 weeks	No
Terrando [22]	C57BL/6	4	12–14 w	M	*E. coli* (O111:B4)	ip	1	1 day	Yes
								3 days	Yes
								1 week	No
Chung [36]	ICR	7	6 w	M	U	ip	1	6 h	Yes
								12 h	Yes
								1 day	Yes
Chen [25]	C57BL/6	4	8–12 w	M	*E. coli* (O55:B5)	ip	1	1 day	No
Laflamme [3]	CD1	U	20–25 gr	M	*E. coli* (O55:B5)	ip	1	U	Yes
Gao [59]	B6C3F1/J	U	7 m	M	*E. coli* (O111:B4)	ip	1	1 day	Yes
								5 months	No
Okuyama [41]	ICR	10	6 w	M	*S. typhimurium*	ip	1	3 days	Yes
Kaushik [60]	BALB/c	U	6–8 w	U	*S. enterica*	ip	5	1 day	Yes
Hwang [61]	C57BL/6	U	11 w	M	*E. coli* (O55:B5)	ip	5	3 days	Yes
Qin [7]	B6:129SF2	U	8 w	M	*E. coli* (O111:B4)	ip	5	2 h	Yes
	C57BL/6	U						3 h	Yes
Sierra [6]	p7.2*fms*-EGFP	U	2 m	B	*S. typhimurium*	ip	5	1 day	Yes
Masocha [62]	C57BL/6	8	8–12 w	U	*E. coli* (O111:B4)	ip	5	4 h	No
								1 day	Yes
								1 year	Yes
Ha [63]	C57BL/6	5	7 w	M	U	ip	5	3 h	Yes
O’Callaghan [40]	C57BL/6	4	8–10 w	M	*E. coli* (O111:B4)	ip	5	1 day	Yes
								3 months	Moderate
Bhaskar [23]	C57BL/6	U	2 m	U	U	ip	1	1 day	Moderate
							10	1 day	Yes
Nishioku [45]	ICR	4	8 w	M	*E. coli* (O55:B5)	ip	20	1 h	No
								3 h	No
								6 h	Yes
								1 day	Yes
Smithason [20]	C57BL/6	6	10–12 w	M	U	ip	200	2 days	Moderate
Sehgal [8]	C57BL/6	15	2–3 m	M	*E. coli* (O55:B5)	sc	3	12 h	Yes
Rats									
Monje [64]	Fisher 344	3	160–180 gr	F	U	ip	1	1 week	Yes
Fan [39]	Sprangue-Dawley	6	5 d	B	*E. coli* (O55:B5)	ip	2	1 day	Yes
Wang [65]	Sprangue-Dawley	3	280–300 gr	M	*E. coli* (O55:B5)	ip	5	1 day	Yes
Semmler [66]	Wistar	4	250–300 gr	M	*E. coli* (O127:B8)	ip	10	4 h	No
								8 h	No
								1 day	Moderate
Semmler [5]	Wistar	5	250–300 gr	M	*E. coli* (O127:B8)	ip	10	1 day	Yes
Garcia-Bueno [67]	Sprangue-Dawley	5	260–340 gr	M	*E. coli* (O55:B5)	iv	0.002	1–3 h	Moderate*
Jiang-Shieh [28]	Wistar	10	200–250 gr	M	*E. coli* (O55:B5)	iv	0.05	2 days	Yes
Buttini [44]	Sprangue-Dawley	2	180–200 gr	M	*E. coli* (O55:B5)	iv	1	1 day	No*
							2.5 and 5	1 h	No*
								3 h	Moderate*
								6 h	Moderate*
								8 h	Yes*
								1 day	Yes*
								3 day	Moderate*
								1 week	No*

Column time of termination is the time from the (first) LPS challenge

Abbreviations: *N* number of animals per group, *m* months, *wk* weeks, *d* days, *gr* gram, *U* unknown, *M* male, *F* female, *B* both sexes, *ip* intraperitoneal, *sc* subcutaneous, *iv* intravenous, *iv* intravenous

*Did not express data in statistical values, no statistical information

Two studies described activation of microglia 6 h after inoculation, 11 of 12 studies (92 %) showed microglial activation 1 day after inoculation, and three studies found microglial activation 3 days after challenge. Findings implicate that a single LPS challenge activates microglia 6 h after challenge and that the activation remains for at least 3 days. Five studies evaluated microglial response after this period: three studies showed no microglial activation after 1 week, 5 weeks, and 5 months, while another study reported moderately activated microglia after 3 months. One study showed microglial activation 1 year after LPS challenge (Table 1). Importantly, overall, the interpretation of the results was hampered by lack of information. The number of animals was noted in only 13 of 20 studies (65 %), and a statistical test comparing microglial response in LPS challenged and control groups were provided in 10 studies (50 %). LPS challenges in experiments using knockout mice were described in four studies, all using intraperitoneal injection, and are discussed below in the separate sections (Appendix) [22–25].

A single LPS challenge in rats was evaluated in eight studies. LPS was given intraperitoneally (63 %) or intravenously (37 %; Table 1), and seven studies used LPS from *E. coli*. LPS origin was not mentioned in one study. Dose of LPS ranged between 0.002 and 10 mg/kg. In general, microglia start to become moderately active 3 h after LPS challenge, reaching their activation state after 8 h to 2 days, and return to their normal resting state after 7 days.

Sequential LPS challenges were evaluated in nine studies (Table 2); seven studies used mice and two studies rats. Five of nine studies (56 %) were done in mice with a C57BL/6 genetic background, and age of these animals varied between 5 weeks and 10 months. LPS from *E. coli* was used in six of the nine studies; origin of LPS was not mentioned in three studies. Dose of LPS ranged from 0.05 to 4 mg/kg. Animals were challenged between 2 and 48 times over a period of 1 day to 6 months with total LPS dose ranging from 0.1 to 56 mg/kg. Eight of these nine studies showed microglial activation (89 %); however, the majority of time points evaluated after 1 month showed only moderate or no microglial activation.

**Table 2:** Multiple intraperitoneal challenges with lipopolysaccharide (LPS) in mice and rats

Study	Genetic background	*N*	Age	Sex	Type of LPS	Dose (mg/kg)	Number of hits	Total dose (mg/kg)	Time of termination	Microglial activation
Mice										
Frank-Cannon [38]	C57BL/6	3	6–13 w	U	*E. coli* (O111:B4)	0.1 b.i.w.	16×	1.6	2 months	No
						0.1 b.i.w.	24×	2.4	3 months	Yes*
						0.1 b.i.w.	48×	4.8	6 months	No
Katafuchi [68]	C57BL/6	8	10 m	M	U	0.25 q.d.	7×	1.75	7 days	Yes
Lee [52]	ICR	5	5 w	M	U	0.25 q.d.	7×	1.75	18 days	Yes
Nguyen [34]	C57BL/6	U	6 m	U	*E. coli* (O55:B5)	1 q.2wk.	6×	6	15 weeks	No*
Franciosi [46]	FVB/N	5	5 m	B	U	1 q.wk.	4×	4	4 weeks	Yes
			8 m				16×	16	17 weeks	Yes
Chen [25]	C57BL/6	4	8–12 w	M	*E. coli* (O55:B5)	1 q.d.	2×	2	3 days	Moderate*
							4×	4	5 days	Yes
Shankaran [69]	C57BL/6	5	10–15 w	F	*E. coli* (O111:B4)	0.3 q.a.d.	4×	1.2	7 days	No
						1 q.a.d.	4×	4	7 days	Yes
						4 q.a.d.	4×	16	7 days	Yes
						4 q.a.d.	14×	56	4 weeks	Yes
Rats										
Yin [70]	Sprangue-Dawley	4	3 d	M	*E. coli* (O55:B5)	0.05 q.a.d	2×	0.1	6 days	Yes
									18 days	Yes
									42 days	No
Wu [9]	Sprangue-Dawley	8	10 w	M	*E. coli* (U)	1.2 q.d.	14×	16.8	1 week	Yes

Column time of termination is the time from the (first) LPS challenge. All challenges were intraperitoneal

Abbreviations: *N* number of animals per group, *U* unknown, *M* male, *F* female, *B* both sexes, *m* months, *w* weeks, *d* days, *gr* gram, *q.d.* every day, *q.2wk* once every 2 weeks, *q.wk* once a week, *q.a.d.* every other day, *b.i.w.* 2 times a week

*Did not express data in statistical values, no statistical information

One study administered LPS 1 mg/kg intraperitoneally in 6-month-old male gerbils, prompting a moderate microglial response at day 4 [26]. Another study evaluated the effect of serial intraperitoneal injections of 0.2 mg/kg LPS at different stages of brain development in opossums (on postnatal day [P] P14, P35, and P42) [27]. At day 10 after the first LPS injection, immunohistochemistry revealed microglial activation in younger age groups (P14 and P35) but not in older animals (P42) [27].

#### Lipoteichoic acid

One study evaluated intravenous administration of 20 mg/kg lipoteichoic acid (LTA) from *Staphylococcus aureus* (strain L2515) in rats [28]. Two days after challenge, microglial cells were activated as shown by enhanced immunoreactivity for CD11b and MHCII as compared to the unchallenged group, 2 days after LTA challenge. In these experiments, CD68 immunoreactivity of the pineal microglia appeared unaltered, while challenge with LPS (0.05 mg/kg) induced enhancement of CD68 immunoreactivity in addition to response for morphology, CD11b, and MHCII expression.

#### Bacteria

Bacteria were used as systemic challenge in 12 of 51 studies (24 %; Table 3). Animal species were rats in the majority of studies (75 %). Live bacteria were used in seven studies (58 %) and heat-killed bacteria in five studies (42 %). *E. coli* (American Type Culture Collection (ATCC) 15746) was most commonly used as systemic challenge with live bacteria (86 %); other live bacteria administered were *Salmonella typhimurium*. The heat-killed bacteria that were used were *Mycobacterium tuberculosis* and *Mycobacterium butyricum*, also referred to as complete Freund’s adjuvant (CFA).

**Table 3:** Challenge with pathogens in mice and rats

Study	Genetic background	N	Age/weight	Sex	Pathogen	Site of challenge	Dose		
Mice									
Püntener [43]	C57BL/6	3	>8 w	F	*S. typhimurium* (SL3261)	ip	1 × 10^6^ CFU	1 day	No
								7 days	Moderate
								21 days	No
Rabchevsky [31]	C57BL/6	6	6 w	F	CFA	sc + ipl	150 ug	14 days	No
								21 days	No
Di Filippo [32]	Biozzi ABH	4	6–8 w	F	CFA	sc + ipl	100 ug	U	Yes
Rats									
Bland [71]	Sprangue-Dawley	8	4 d	M	*E. coli* (ATCC 15746)	sc	1 × 10^5^ CFU/g	2 days	Yes
								70 days	Yes
								98 days	Yes
Williamson [72]	Sprangue-Dawley	9	4 d	M	*E. coli* (ATCC 15746)	sc	1 × 10^5^ CFU/g	2–3 months	Yes
Bilbo [73]	Sprangue-Dawley	6	4 d	M	*E. coli* (ATCC 15746)	sc	1 × 10^5^ CFU/gram	2 months	No*
								16 months	Yes
Bilbo [42]	Sprangue-Dawley	8	4 d	M	*E. coli* (ATCC 15746)	sc	1 × 10^5^ CFU/g	56 days	Yes
Bilbo [35]	Sprangue-Dawley	6	4 d	M	*E. coli* (ATCC 15746)	sc	1 × 10^5^ CFU/g	56 days	No
Bilbo [37]	Sprangue-Dawley	8	4 d	M	*E. coli* (ATCC 15746)	sc	1 × 10^6^ CFU	2 h	No
								8 h	No
								1 day	Yes
								2 days	No
								3 days	Yes
								2-3 months	Yes
Raghavendra [4]	Sprangue-Dawley	4	175–200 gr	M	CFA	ipl	100 ul	4 h	Moderate
								4 days	Yes
								2 weeks	Yes
Liu [30]	Lewis	6	2 m	F	Heat-killed *M. butyricum*	id	1.5 mg	3 weeks	Moderate*
			12 m						Yes
Wu [29]	Lewis	6	100–110 gr	F	Heat-killed *M. butyricum*	id	25 mg/kg	2 weeks	Yes
								3 weeks	Yes
								4 weeks	Yes

Column time of termination is the time from the (first) LPS challenge

Abbreviations: *U* unknown, *F* female, *m* months, *d* days, *ip* intraperitoneal, *ipl* intraplantar, *M* male, *B* both sexes, *w* weeks, *gr* gram, *sc* subcutaneous, *id* intradermal, *N* number of animals per group, *CFA* complete Freund’s adjuvant

*Did not express data in statistical values, no statistical information

Six studies from one research group focused on the effect of systemic infectious challenge in early life. In these studies, infant rats (P4) were challenged with *E. coli* 1x 10^5^ colony-forming units (CFU)/g subcutaneously. Immunohistochemistry with Iba-1 revealed activated microglia in the hippocampus 2 days after challenge and increased CD11b expression in the hippocampus area up to 3 months after infection. Another study using *S. typhimurium* (SL3261; 10^6^ CFU) showed increased expression of CD11b and CD68 in the thalamus 7 days after the challenge. Interestingly, CD11b and CD68 expression returned to baseline levels 3 weeks after challenge. At all time points during this experiment, microglia were morphological ramified with fine processes.

Studies evaluating challenge with heat-killed bacteria used CFA, a solution composed of heat-killed and dried mycobacteria (usually *M. butyricum* and/or *M. tuberculosis*). Injecting CFA intradermal or intraplantar induces adjuvant arthritis, a model of chronic peripheral inflammation. One study showed CD11b messenger ribonucleic acid (mRNA) expression in brainstem and forebrain 4 h, 4 days, and 2 weeks after CFA challenge [4]. Another study challenging rats with 25 mg/kg CFA showed cortical microglial activation immunohistochemistry with Iba-1 2, 3, and 4 weeks after inoculation [29]. One study evaluated the role of age and compared rats of two and 12 months old, using 1.5 mg challenge of CFA, showing morphologically activated microglia revealed in the CA1 region of the hippocampus for both age groups, three weeks after challenge [30]. However, no CD68 or IL-1β-positive microglial cells were detected in the brains of 2-month-old rats, while the expression of CD68 and IL-1β was significantly increased in hippocampal CA1 region of 12-month-old rats compared to control rats.

CFA is also used to induce experimental allergic encephalomyelitis (the EAE model), an animal model for multiple sclerosis (MS), by peripheral injections of CNS tissue homogenized with these heat-inactivated mycobacteria. Only the control groups (CFA) could be included in the current review. One mouse study showed no microglial activation in the brainstem up to 3 weeks after inoculation with 150 ug CFA [31]. Another mouse study using 100 ug CFA on days 1 and 7 showed activated microglia in the CA1 region of the hippocampus 22 days after the last challenge [32].

#### Inflammatory parameters

Inflammatory parameters were reviewed if they were evaluated concomitantly with the microglial response. This was done in 23 of 51 studies (45 %).

#### Toll-like receptors

Seven studies evaluated TLRs on microglia, by qPCR, immunohistochemistry, in situ hybridization, or flow cytometry: four evaluated TLR-2 expression [3, 21, 33, 34] and three TLR-4 expression [4, 35, 36]. Three studies described TLR-2 upregulation [3, 21, 33] and two TLR-4 upregulation [4, 36]. Microglial activation was associated with TLR upregulation independent of type of challenge (LPS, *E. coli*, or CFA) or time point of evaluation. In those studies showing resting microglia after challenge, TLR expression was not different from the control group. One study challenged TLR-4 knockout mice with serial LPS injections of 1 mg/kg every day for 4 days, showing decreased microglial activation in the knockout mice as compared to wild-type animals (Appendix) [25]. Although TLR-2 is known for its recognition of lipopeptides, peptidoglycans (PGN), and LTA, all of which are cell wall components of gram-positive bacteria, TLR-2 upregulation was found after LPS challenge [3, 21, 33]. One study using a head-to-head comparison between LPS, PGN, and LTA challenges showed a profound transcriptional activation of TLR-2 only after LPS.

#### Cytokines and chemokines

TNF-α protein levels or mRNA expression were determined in 14 studies [3–9, 29, 32, 34, 37–40], by qPCR, ELISA, western blot, in situ hybridization, and immunohistochemistry, at 23 different time points after the challenge. Microglial activation was associated with increased expression of TNF-α in the brain, as compared to controls, at 12 time points: ranging from 3 h to 1 day after single LPS (*n* = 5), after multiple LPS (*n* = 1), or after a CFA challenge (*n* = 6). At four time points, microglial activation was described without increased TNF-α protein levels: 1 day after single LPS (*n* = 1), 1 and 3 days after *E. coli* (*n* = 2), or 22 h after CFA challenge (*n* = 1). One study observed an elevation of TNF-α mRNA and protein levels 30 min after a single LPS challenge [7], and this effect remained up to 10 months after the LPS administration. Interestingly, the studies evaluating *E. coli* challenge showed no differences in TNF-α concentration as compared to the control group, independent of whether or not microglia were activated [37].

Interleukin 1 beta (IL-1β) protein levels or mRNA expression were determined in 16 studies [4–6, 8, 9, 21, 22, 30, 32, 35, 37, 39–43], at 24 different time points after challenge. At 13 time points microglial activation was present in combination with increased IL-1β protein levels or mRNA expression patterns, from 4 h to 1 week after LPS challenge (*n* = 7), from 1 and 56 days after *E. coli* challenge (*n* = 2), and from 4 h to 3 weeks after CFA challenge (*n* = 4). At three time points, microglia were activated but no IL-1β increase was measured; 1 day after LPS (*n* = 1), 2 days after *E. coli* (*n* = 1), and 22 h after CFA challenge (*n* = 1). In studies showing microglia in resting or moderately activated states, brain IL-1β levels were comparable to that of the control group (*n* = 7). IL-1 receptor (IL-1R) knockout mice were used in one study, evaluating a single dose of 1 mg/kg of LPS (Appendix) [22], and IL-1R knockouts had no microglial activation in the CA1 region of the hippocampal area, in contrast to the wild-type group. IL-1R antagonist (IL-1Ra) injection in wild-type mice just before the LPS challenge also prevented microglial activation, suggesting an important role of IL-1 in the activation of microglia.

Interleukin 6 (IL-6) protein levels or mRNA expression were determined in seven studies [4–6, 8, 9, 22, 37], at 17 different time points after challenge. At five time points, microglial activation was present in combination with increased protein levels or mRNA expression patterns, at 12 h and 1 week after a single LPS (*n* = 2) and at 4 h, 4 days, and 2 weeks after CFA challenge (*n* = 3). At five time points, microglia were activated but brain IL-6 levels were similar to controls: 1 day after single LPS (*n* = 3) and 1 and 3 days after *E. coli* challenge (*n* = 2). Animals challenged with *E. coli* had similar IL-6 concentration as compared to the controls, independent of whether microglia were activated (*n* = 2) or not (*n* = 3).

Interleukin 10 (IL-10) protein levels or mRNA expression were determined in seven studies [5, 6, 8, 21, 29, 37], at 12 different time points after challenge. At four time points, microglia were activated in combination with increased protein levels or mRNA expression patterns: 4 h and 1 day after systemic LPS (*n* = 2) and 3 and 4 weeks after CFA challenge (*n* = 2). At four time points, microglia were activated but brain IL-10 levels were similar to controls: 1 day after LPS (*n* = 1), 1 and 3 days after *E. coli* (*n* = 2), and 2 weeks after CFA challenge (*n* = 1). One study showed microglial activation after LPS challenge but decreased levels of IL-10 in the brain [8].

Two studies measured transforming growth factor beta (TGF-β) mRNA expression with qPCR 1 day after a single LPS challenge [5, 6]. Microglial cells were activated in both studies. One study measured brain TGF-β mRNA expression and found increased concentrations [5]. The other study measured TGF-β in isolated microglia and found no differences between challenged and control groups [6]. Two studies measured monocyte chemotactic protein 1 (MCP-1) mRNA expression in the brain [5, 8], showing that microglial activation was associated with MCP-1 upregulation. Fractalkine receptor (CX3CR1) knockout mice were used in two studies evaluating a single dose of 10 mg/kg LPS [23] and multiple LPS challenges (20 ug for 4 days) (Appendix) [24]. One day after the (last) LPS challenge, immunohistochemistry with Iba-1 revealed enhanced microglial activation in the hippocampal area of the CX3CR1 knockout mice as compared to wild-type animals.

#### Blood–brain barrier

The blood–brain barrier (BBB) was examined in eight studies [27, 28, 31, 36, 37, 44–46]. Four studies showed disruption of the BBB (challenge was LPS in two studies, either intraperitoneal or intravenous, *E. coli* subcutaneously in one study and CFA intraperitoneal in the other study) [28, 31, 37, 45], three studies showed intact BBB (challenge was LPS in all three studies, either intraperitoneal [*n* = 2] or intravenous [*n* = 1]) [36, 44, 46], and one study showed inconclusive results (challenge was LPS) [27]. Studies were highly variable with respect to methods used to define the integrity of the BBB (fluorescent sodium, protein and fibrinogen extravasation, exogenous horseradish peroxidase, Evans blue dye, ribosomal ribonucleic acid (rRNA) of *E. coli*, fluorescent LPS, and the invasion by blood monocytes or macrophages).

## Discussion

Experimental studies have shown that peripheral inflammatory stimuli, such as LPS, cause a profound immunological response in the brain resulting in microglial activation. After a single challenge of LPS, microglia were moderately active within 3 h after administration, reaching their profound activation state after 8 h to 2 days and subsequently return to their normal resting state after 7 days. Interestingly, cytokine expression levels in the brain and activation markers may remain elevated for months after a single LPS challenge. These experiments also showed that systemic challenge with live bacteria, mainly the gram-negative bacteria *E. coli* and *S. typhimurium*, causes microglial activation. A gram-negative bacterium contains approximately 10^−15^ g of LPS, which implicates that the LPS dosages used in the included animal studies are supernatural. Consequently, the microglial response of challenge with bacteria is less profound as compared to that found in the experiments using a challenge with supernatural LPS doses. LPS as a peripheral inflammatory challenge is much easier to use than live bacteria: there is no waiting for bacteria to be cultured, no monitoring of bacteria to grow in midlog phase, and no time is lost with harvesting, wash and dilute the bacteria in the right amount. In addition, there is no danger of contamination, and therefore, the laboratory and animal facility do not have to comply with special safety matters. However, because of the differences in microglial response between a peripheral challenge with LPS or live bacteria, the clinical relevance of using LPS in these animal models is questionable. Experimental studies using live bacteria suggest an important role of age in the process of microglial activation after systemic challenge, although further research is needed to confirm this. The observed effects support the central role of microglial response in the development of sepsis-associated delirium and poor functional outcome after sepsis, in particular in the elderly population [15].

The mechanisms connecting systemic inflammatory challenge and microglial activation remain unclear. Several pathophysiological mechanisms have shown to play a role in this process. Microglia may be activated through primary autonomic afferents—in particular the vagal nerve—by active BBB transport of pro-inflammatory chemo- and/or cytokines, passive transport of pro-inflammatory products via the circumventricular organs, or by signaling the epithelial cells of the blood–brain barrier [47]. Microglial activation was associated with upregulation of TLRs, independent of type of challenge or time point of evaluation. In studies in which microglial activation was not observed, TLR expression was not different from the control group [34, 35]. Moreover, microglia could not be activated in TLR receptor knocked-out mice [25]. Microglial activation was associated with elevated levels of pro-inflammatory mediators in the brain.

Age is an important intrinsic factor determining the level of microglial activation after a systemic inflammatory challenge. The normal aging process induces changes in microglial phenotype, and these age-related changes are also called “priming” [48]. Two studies compared the effect of age on microglia after a peripheral challenge [21, 30]. Systemic LPS challenges caused a hyperactive microglial response in the brain of aged mice, associated with higher induction of inflammatory IL-1β and anti-inflammatory IL-10 [21]. Peripheral CFA injection induced hippocampal microglial activation in middle-aged rats and moderate activation in young rats. In these experiments, microglial activation in middle-aged rats was associated with neurocognitive deficits [30]. Aging-induced immune senescence occurs in the brain as age-associated microglial senescence, which renders microglia to function abnormally and may eventually promote neurodegeneration.

Evidence suggests that microglia act neurotoxic when fully activated (M1 phenotype) [49], whereas other studies show that activated microglia show more diversity and can have a role in remodeling and repair as well (M2 phenotype) [50, 51]. The M1 immune response of microglia is triggered by the activation of TLRs via pathogen-associated molecular patterns (PAMPs) or intracellular proteins released from damaged neurons; other M1 triggers are complement 1q (C1q) and adenosine triphosphate (ATP) released from astrocytes in response to neuronal injury [11, 12]. These activated M1 microglia produce the pro-inflammatory mediators TNF-α, IL-1β, and IL-6 [11, 12]. M2 activation is induced by signals from apoptotic cells that activate triggering receptor expressed by myeloid cells-2 (TREM2) such as heat shock protein 60 (Hsp60), or by anti-inflammatory cytokines, such as interleukin 14 (IL-14) and interleukin 13 (IL-13). M2-activated microglia have a role in remodeling and repair, triggering anti-inflammatory responses via release of TGF-β and IL-10 [11–13]. While beyond the scope of this review, several studies show an association between systemic LPS challenge, microglial activation, and cognitive deficits in mice [52–55]. These studies demonstrate that systemic LPS challenge causes cyclooxygenase-1 (COX-1), COX-2, and inducible nitric oxide synthase (iNOS) expression in the brain, which is hypothesized to cause susceptibly to cognitive deficits in mice [52, 53, 55]. Regarding these facts, it is imperative—when it comes to defining microglial activation—to focus not only on proliferation and morphology but also examine pro- and anti-inflammatory markers on or around microglia. If more homogeneous data on these inflammatory markers, in relation to microglial activation, would be available, then bigger steps can be made in understanding the pathogenesis in why neuro-inflammation occurs when a systemic challenge is administrated. However, less than half of the studies (45 %) included in this review contain data on inflammatory mediators. When microglia were activated, an increase in TLR-2, TLR-4, TNF-α, and IL-1β mRNA expression or protein levels in the brain was seen in most studies. A few studies examined anti-inflammatory markers IL-10 (*n* = 6), TGF-β (*n* = 2), and MCP-1 (*n* = 2).

Studies have shown that microglial cells express various neurotransmitter receptors [56], and neurotransmitters could also exert pro- and anti-inflammatory effects on microglial cells. For example, ionotropic glutamate receptors (iGluRs) can modulate TNF-α release, gamma-amino-butytric acid (GABA) receptors modulate IL release (IL-6 and IL-12) and adrenergic, dopaminergic, and cholintergic receptors exhibit anti-inflammatory effects [57]. These receptors add another challenge on the pathogenesis on neuro-inflammation and should not be ignored.

Heterogeneity among the included 51 studies hampered the opportunity for a synthesis, e.g., quantitatively, in this systematic review. Studies investigated several animal species and inflammatory stimuli at different time points. Lack of adequate experimental description, power calculations for animal group sizes, reported baseline measurements of animals between groups, or handling of outlined or missing data further limited generalization of the results. Nevertheless, this review provides a valuable overview of current knowledge.

## Conclusion

Experimental studies have shown that peripheral challenge with LPS causes a profound immunological response in the brain resulting in microglial activation, but systemic challenge with live bacteria causes microglial activation as well. However, the microglial response of challenge with bacteria is less profound as compared to that found in the experiments using a challenge with supernatural LPS doses. The mechanisms connecting systemic inflammatory challenge and microglial activation remain unclear, but age is an important intrinsic factor determining the level of microglial activation after a systemic inflammatory challenge. Heterogeneity among the included studies hampered the opportunity for a synthesis, e.g. quantitatively, in this systematic review. Future experimental studies should opt for mouse models, use live bacteria as well as standardized and quantitative measurements of microglial activation, for example, with flow cytometry, and focus on the role of the aging brain. These studies should apply with the current standards of animal experiments [19]. For optimal external validation, experimental studies should investigate the role of aging on microglial activation following a systemic infection with live bacteria, analogous to the human situation: the clinical problem of long-term poor outcome of sepsis-associated delirium in elderly patients (Table 4).

**Table 4:** Keypoints review

Keypoints
• Systemic challenge with LPS and live bacteria cause a profound immunological response in the brain resulting in microglial activation.
• Microglial response after challenge with bacteria is less profound as compared to challenge with LPS, which makes the clinical relevance of using LPS in these animal models questionable.
• When defining microglial activation, researcher should not only focus on proliferation and morphology, but also examine pro-and anti-inflammatory markers indicating M1/M2 responses.
• Mechanisms connecting systemic inflammatory challenge and microglial activation remain unclear, but age is an important intrinsic factor.
• Future experimental studies on studying systemic inflammation and microglial response should do the following: −use mouse models −use live bacteria −use standardized and quantitative measurements of microglial activation −focus on the role of the aging brain −apply with the current standards of animal experiments

## Appendix

**Table 5:** Intraperitoneal challenge with lipopolysaccharide (LPS) in knock-out mice

Study	Knock out gene	*N*	Age	Sex	Type of LPS	Dose	Time of termination	Microglial activation	Compared to wild-type
Bhaskar [23]	CX3CR1^−/−^	U	2 m	U	U	10 mg/kg	1 d	Yes*	More*
Terrando [22]	IL-1R^−/−^	4	12–14 w	M	*E. coli* (O111:B4)	1 mg/kg	1 d	No	Less*
							3 d	No	Less*
							1 w	No	Same*
Cardona [24]	CX3CR1^−/−^	U	U	U	U	20 ug q.d. 2×	4 d	Yes*	More*
Chen [25]	TLR4^−/−^	4	8–12 w	M	*E. coli* (O55:B5)	1 mg/kg q.d. 2×	4 d	No*	Less*

Column time of termination is the time from the (first) LPS challenge. All challenges were intraperitoneal

Abbreviations: *U* unkown, *m* months, *d* days, *M* male, *w* weeks, *q.a.d* every other day, *N* number of animals per group

*Did not express data in statistical values, no statistical information

## Abbreviations

ATCC: American Type Culture Collection
ATP: adenosine triphosphate
BBB: blood–brain barrier
C1q: complement 1q
CD11b: cluster of differentiation 11b
CD45: cluster of differentiation 45
CD68: cluster of differentiation 68
CFA: complete Freund’s adjuvant
CFU: colony-forming units
COX-1: cyclooxygenase-1
COX-2: cyclooxygenase-2
CX3CR1: fractalkine receptor
*E. coli*: *Escherichia coli*
GABA: gamma-amino-butytric acid
Hsp60: heat shock protein 60
IB4: *Griffonia symplicifolia* isolectin B4
Iba-1: ionized calcium-binding adaptor molecule 1
iGluRs: ionotropic glutamate receptors
IL-10: interleukin 10
IL-13: interleukin 13
IL-14: interleukin 14
IL-1R: interleukin-1 receptor
IL-1Ra: interleukin-1 receptor antagonist
IL-1β: interleukin 1 beta
IL-6: interleukin 6
iNOS: inducible nitric oxide synthase
LPS: lipopolysaccharide
LTA: lipoteichoic acid
*M. butyricum*: *Mycobacterium butyricum*
*M. tuberculosis*: *Mycobacterium tuberculosis*
MARCO: macrophage receptor with collagenous structure
MHCII: major histocompatibility complex II
MCP-1: monocyte chemotactic protein 1
mRNA: messenger ribonucleic acid
MS: multiple sclerosis
P: postnatal day
PAMPs: pathogen-associated molecular patterns
PGN: peptidoglycans
qPCR: quantitative polymerase chain reaction
rRNA: ribosomal ribonucleic acid
*S. typhimurium*: *Salmonella typhimurium*
TGF-β: transforming growth factor beta
TLR: Toll-like receptor
TLR-2: Toll-like receptor 2
TLR-4: Toll-like receptor 4
TLRs: Toll-like receptors
TNF-α: tumor necrosis factor alpha
TREM2: triggering receptor expressed by myeloid cells-2
